# The Overlooked Biomechanical Role of the Clasping Leaf Sheath in Wheat Stalk Lodging

**DOI:** 10.3389/fpls.2021.617880

**Published:** 2021-08-20

**Authors:** Joseph Cornwall, Christopher J. Stubbs, Christopher S. McMahan, Daniel J. Robertson

**Affiliations:** ^1^Department of Mechanical Engineering, University of Idaho, Moscow, ID, United States; ^2^School of Mathematical and Statistical Sciences, Clemson University, Clemson, SC, United States

**Keywords:** leaf sheath, lodging, stalk, stem, bending, strength, wheat, biomechanics

## Abstract

The biomechanical role of the clasping leaf sheath in stalk lodging events has been historically understudied. Results from this study indicate that in some instances the leaf sheath plays an even larger role in reinforcing wheat against stalk lodging than the stem itself. Interestingly, it appears the leaf sheath does not resist bending loads by merely adding more material to the stalk (i.e., increasing the effective diameter). The radial preload of the leaf sheath on the stem, the friction between the sheath and the stem and several other complex biomechanical factors may contribute to increasing the stalk bending strength and stalk flexural rigidity of wheat. Results demonstrated that removal of the leaf sheath induces alternate failure patterns in wheat stalks. In summary the biomechanical role of the leaf sheath is complex and has yet to be fully elucidated. Many future studies are needed to develop high throughput phenotyping methodologies and to determine the genetic underpinnings of the clasping leaf sheath and its relation to stalk lodging resistance. Research in this area is expected to improve the lodging resistance of wheat.

## Introduction

Wheat stalk lodging—the permanent displacement of plants from their upright position—results in millions of dollars of lost revenue each year (Berry and Spink, 2012; USDA, 2013). Furthermore, lodging is a primary constraint to increasing the yield of current wheat varieties (Berry et al., 2003a, 2004). Lodging can be categorized into two types: stalk lodging and root lodging. Root lodging occurs when the plant becomes uprooted or the roots break (Stubbs et al., 2019a). Stalk lodging occurs when the stem of the plant breaks (Robertson et al., 2016, 2017; Stubbs et al., 2019b, 2020b). Numerous studies of lodging resistance, root anchorage strength, and stalk strength have been conducted previously in an effort to alleviate the problem of wheat stalk lodging (Berry et al., 2003b, 2004, 2007; Berry and Spink, 2012). These studies have focused on the role of chemical composition (Berry et al., 2003a; Kong et al., 2013), morphology (Keller et al., 1999; Zuber et al., 1999; Tripathi et al., 2002, 2003), weather (Easson et al., 1993; van der Velde et al., 2012; Mäkinen et al., 2018), disease (Loyce et al., 2008), and pests (Daamen et al., 1989). In general, these studies have investigated stalk strength (defined herein as the bending strength of the stem-sheath complex) or have removed the leaf sheath and investigated stem strength directly. However, the biomechanical role of the clasping leaf sheath has rarely been investigated directly. In particular, the authors are only aware of a single case study in which the biomechanical role of the leaf sheath of wheat was investigated directly (Wu and Ma, 2019). Consequently the mechanical role of the leaf sheath is not fully understood (Wu and Ma, 2019).

**Figure 1:**
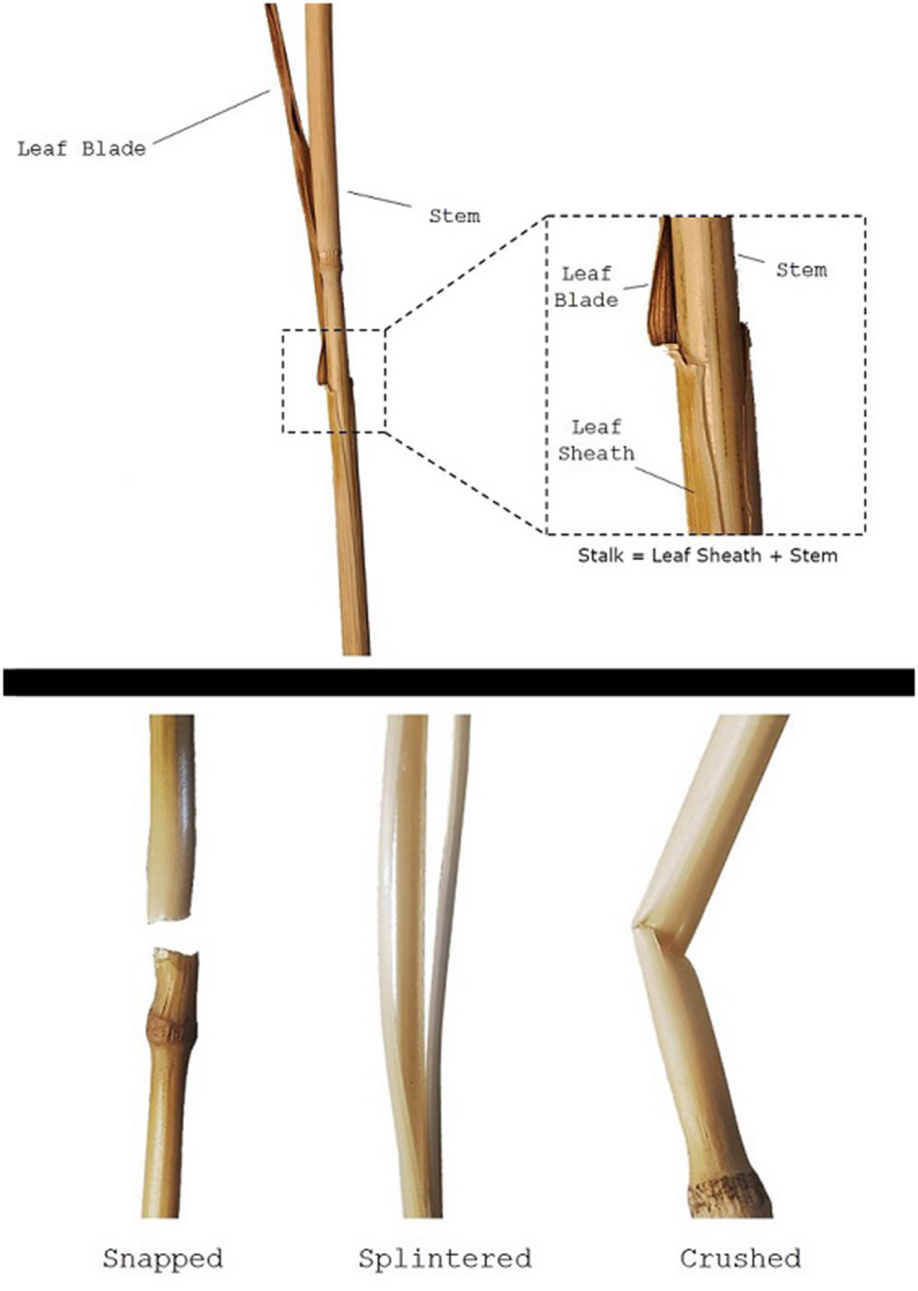
A wheat stem specimen, showing the stem, leaf blade, and leaf sheath (top panel). Note the stalk is defined in this study as the stem-sheath complex. Failure patterns observed during testing (bottom panel).

The clasping leaf sheath is the portion of the leaf that tightly wraps around the stem before branching outwards into leaf blades (see Figure 1). The sheath plays a critical role in the structural integrity of plants throughout their lifecycle. During growth and development, the leaf sheath supports the stem, and removal of the leaf sheath during early growth often causes the plant to immediately become structurally unstable (i.e., to fall over). While the biomechanical significance of the leaf sheath has been quantitatively investigated in other plant species such as sorghum (Bashford et al., 1976; Niklas, 1998), there is little quantitative research on the biomechanical role of the leaf sheath in wheat stalk lodging events (only a single case study in the last 100 years of research on wheat stalk lodging). Consequently, little is known about the biomechanical role of the leaf sheath. This lack of understanding impedes the creation of more lodging resistant crop varieties.

The only quantitative investigation of the wheat leaf sheath reported in the literature indicated that the sheath increased stalk lodging resistance by increasing the effective moment of inertia of the stalk (i.e., by increasing stalk girth) (Wu and Ma, 2019). However, our lab has qualitatively observed that when fully mature wheat is subjected to bending loads that induce stalk lodging the leaf sheath unravels and becomes separated from the stem. It is typically at the moment when the sheath becomes separated from the stem that the stem breaks. Observation of this phenomenon led us to hypothesize that the leaf sheath does not reinforce the stem by merely increasing the moment of inertia. Rather a number of complex interactions between the stem and sheath may be at play and the leaf sheath may reinforce the stem against buckling.

The purpose of this study is to further quantify the biomechanical role of the clasping leaf sheath in fully mature winter and spring wheat. The effect of the leaf sheath on the plant's flexural rigidity, bending strength, and mechanism of structural failure is investigated. This study builds upon the preliminary results presented in Wu and Ma (2019). Given limited resources the authors purposefully choose an experimental design that did not include replication, but rather choose to increase the number of genotypes and environments included in the study. In other words the authors were interested in discovering universal principles as opposed to identifying genotype or environment specific effects.

## Materials and Methods

### Plant Materials

Forty-five hybrids of hard and soft spring wheat were grown during the 2018/2019 season, and 31 different hybrids of hard and soft wheat were grown during the 2019/2020 season. Plants from the 2018/2019 season were grown in a different field than the plants grown in the 2019/2020 season. In both years plants were sampled from ongoing variety trial experiments and were selected to generally represent typical commercial varieties of wheat. Each plot included in this study contained a unique genotype. In the statistical analyses presented below we controlled for genotype, however this variable aggregates genotypic, plot level and year differences. In both years, plants were grown at University of Idaho facilities with a planting density of 108.5 g seed/plot. All plots were 1.5 x 6 m and contained 6 rows. Fertilizer was broadcast and incorporated by tillage prior to planting. Fertilizer was applied at a rate of 42.2 kg nitrogen, 13.6 kg phosphorus, and 13.6 kg of sulfur per acre. All stalks used in this study were allowed to reach full maturity and remained in the field until harvest, at which point the stalk was collected for testing. Samples were collected at harvest time as opposed to during grain fill (when lodging is more likely to occur) because the authors wanted to determine the minimum expected effect of the leaf sheath. At grain fill or prior to full maturity / senesce the effect of the leaf sheath will be more significant than at harvest time. Forty consecutive stalks from the middle of each plot were cut just above ground level and the most basal 30 cm of the plants were transported to the lab. All stalks were sampled from the middle four rows of the plot and at least 0.5 m from the end of the plot to minimize border effects on stalk growth (Watson and French, 1971). Only stalks that were found to be free of disease and mechanically in good condition were included in the study. All samples were stored in a testing room maintained at standard room temperature and humidity after collection (~20°C and ~15%−30% relative humidity).

### Measuring Stalk Flexural Rigidity and Bending Strength—Unpaired Data

**Figure 2:**
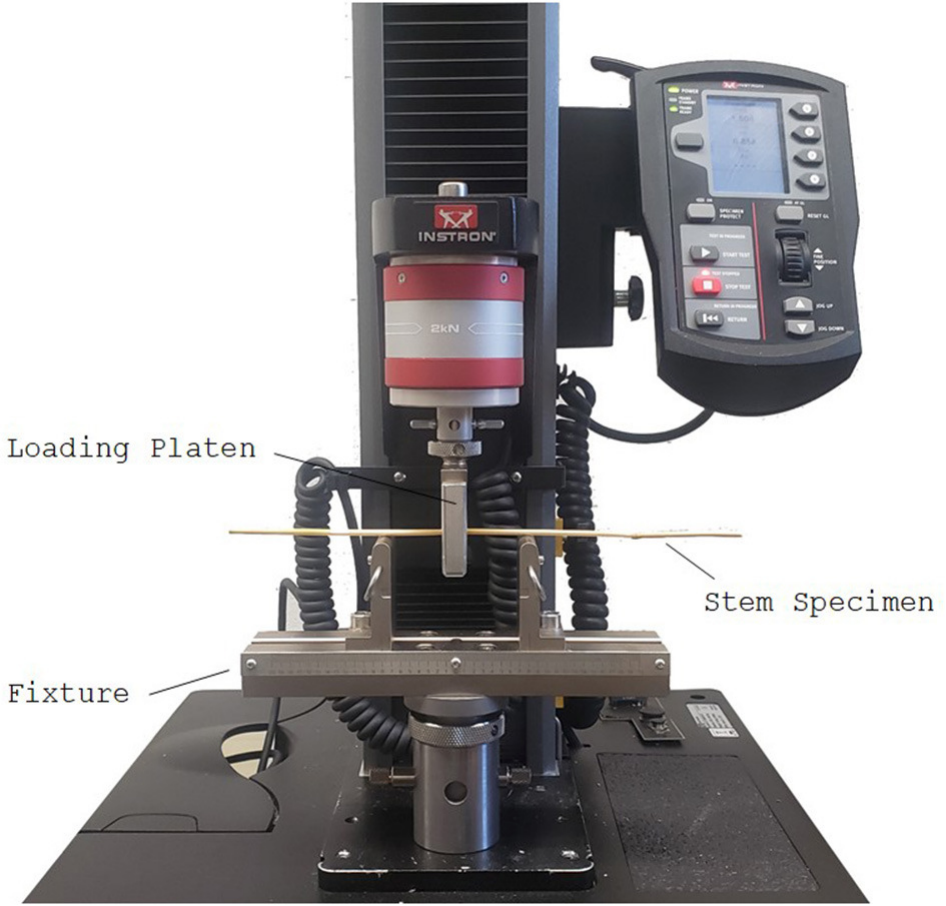
Long span three-point bending test setup of a wheat specimen using an Instron universal testing machine. When testing grasses in three-point bending it is important to load the sample at a node as shown and to utilize long span lengths (>10 x diameter of the sample) to prevent premature failure and transverse crushing of the cross-section (Robertson et al., 2014, 2015b; Stubbs et al., 2018).

Three-point bending tests were performed to measure the effect of the leaf sheath on stalk flexural rigidity and stalk bending strength. From each plot, eight to 10 specimens with a leaf sheath and eight to 10 specimens without a leaf sheath were tested in three-point bending to failure. As measuring bending strength is a destructive test, the specimens analyzed were unpaired, i.e., the specimens with a sheath were different physical specimens than the ones without a sheath. When removing the leaf sheath from the stem special care was taken to ensure the stem was not damaged. In cases where the stems were visually determined to be especially prone to damage, a razor blade was used to mitigate inadvertent loading of the wheat stem during sheath removal. Any stems that were damaged during leaf sheath removal were excluded from the study. All tests were performed using an Instron universal testing machine (Model 5965, Instron Corp., Norwood, MA). The testing protocol was based on previous protocols developed in our lab for maize (Robertson et al., 2014, 2015b; Al-Zube et al., 2018), with the following modifications: (1) A custom fixture was developed to test the specimens with an 8 cm span length (the length from the left-most simple support to the right-most simple support, as shown in Figure 2), with the load being applied to the node closest to the bottom of each stem sample (see Figure 2). The span length was chosen to ensure proper specimen aspect ratios (length/diameter) to minimize transverse deformation of the stalk's cross-section (Stubbs et al., 2018). (2) The stalk was displaced at a constant rate of 2 mm/min until failure occurred. (3) A 50-N load cell was used to collect force data at a rate of 100 ms. The maximum bending moment (M) was calculated at failure by multiplying the maximum applied load (F) by the specimen length (L) and dividing by 4 (Young and Budynas, 2002; Beer et al., 2012):

(1) $$\begin{array}{r} M=\;FL/4 \end{array}$$

The flexural rigidity (EI) of each specimen was calculated with the applied load (F), the span length (L), and the deflection of the specimen (δ) using the Euler-Bernoulli beam equation for three-point bending (Young and Budynas, 2002; Beer et al., 2012):

(2) $$\begin{array}{r} EI=\;\frac{FL^{3}}{48\delta } \end{array}$$

### Failure Pattern Analysis—Unpaired Data

Failure patterns, the observable result of a structure failing due to a specific failure mode, can be used to gain insight into which failure mode—and therefore what geometric and material parameters—are influencing the strength of the wheat stalks (Robertson et al., 2015a). When conducting three-point bending tests the three failure patterns shown in Figure 1 were consistently observed: snapped, splintered, and crushed. Snapped stems have a clean break at the node leaving the stem in two separate pieces. Splintered stems split longitudinally. Crushed stems creased and collapsed near the node through Brazier buckling (Schulgasser and Witztum, 1992; Wegst and Ashby, 2007; Stubbs et al., 2019b). This failure pattern analysis was based on a protocol developed previously in our lab for maize (Robertson et al., 2015a). The failure pattern was recorded for all plants tested: both those with a leaf sheath and those without. This was done so that the effect of the leaf sheath on failure patterns could be determined.

### Moment of Inertia and Flexural Rigidity Testing—Paired Data

After the destructive three-point bending tests described above were conducted several additional non-destructive bending test were conducted. In particular, non-destructive three-point bending tests were performed on an additional eight to 10 specimens from each plot. These tests were performed in the same manner as previously described, except that the stalk was not loaded until failure. Rather each stalk was displaced by 2 mm at a constant rate of 1 mm/min for 10 cycles. The specimens were first tested with the leaf sheath intact. The leaf sheaths were then carefully removed, and the same specimens were retested again. These paired tests were conducted to more precisely determine the effect of the leaf sheath on stalk flexural rigidity. The maximum displacement of 2 mm was chosen based on preliminary testing. In particular, preliminary tests demonstrated that stalks remained well within the elastic range when deflections were 2 mm or less. In other words, no structural damage or material plasticity occurred when testing stalks (with the leaf sheath intact) that would affect the results of the later tests performed on the stem (with the leaf sheath removed).

Flexural rigidity (EI) is comprised of two terms: the stiffness of the tissue (E), and the bending resistance of the geometry, called the moment of inertia (I). As the wheat sheath adds additional material to the outside of the stem, it will increase the moment of inertia, and therefore increase the bending resistance of the stem. To calculate the moment of inertia, the radius of the stem (R), rind thickness (t), and leaf sheath thickness (s) was measured using calipers. Care was taken to ensure the calipers did not compress the specimen when taking morphological measurements. In addition, the same single individual performed all the morphological measurements to eliminate inter-user measurement variability. The moment of inertia was then calculated both for the stalk (i.e., the stem-sheath complex: Equation 3) and for the stem (i.e., with the leaf sheath removed: Equation 4).

(3) $$\begin{array}{r} I_{w/o\;sheath}=\;\frac{\pi }{4}\left(R^{4}-{\left(R-t\right)}^{4}\right) \end{array}$$

(4) $$\begin{array}{r} I_{w/sheath}=\;\frac{\pi }{4}\left({\left(R+s\right)}^{4}-{\left(R-t\right)}^{4}\right) \end{array}$$

This analysis was conducted to investigate if the leaf sheath increases the bending resistivity of the stem simply because it increases the effective bending geometry of the stem (i.e., moment of inertia) or if there is a more complex strengthening mechanism in play.

### Statistical Analyses: Effect of the Leaf Sheath on Bending Strength and Flexural Rigidity

Statistical analyses were performed on both the 2018/2019 season and 2019/2020 season data. For each analysis, the figures for the 2018/2019 season data are shown herein, and the corresponding figures for the 2019/2020 season data are provided in the Supplementary Material. In both years the data was heteroscedastic. Statistical test of significance used in Analysis of Variance and in multiple regression analyses used to make statistical inferences assume homoscedasticity. Therefore, a square root transformation was applied to the data to ensure model assumptions were meet. After these transformations, all statistical models passed standard diagnostic checks, i.e., residual plots, QQ-plots, normality checks, etc. All statistics were therefore performed on the transformed data. It should be noted that several studies including those conducted by the authors of this manuscript have shown a linear relationship between flexural rigidity and bending strength. That same linear relationship exists in the data collected in this study. However, as the aim of this study was to make statistical inferences regarding the contribution of the leaf sheath on stalk flexural rigidity and stalk bending strength the data was transformed. In particular, a series of multiple linear regression models were fit to the transformed data to quantify the effect of the leaf sheath on stalk bending strength and stalk flexural rigidity. These models take the following form:

(5) $$\begin{array}{r} Y_{i}=\beta_{0}+\beta_{1}X_{i1}+\beta_{2}X_{i2}+\epsilon_{i} \end{array}$$

(6) $$\begin{array}{r} Y_{i}=\beta_{0}+\beta_{1}X_{i1}+\sum \alpha_{j}G_{ij}+\epsilon_{i} \end{array}$$

where *Y*_*i*_ is the response variable (i.e., the square root of bending strength or the square root of flexural rigidity) measured on the *i*th observation, β_0_ is the usual intercept parameter, *X*_*i*1_ is a binary variable taking value 1 if the sheath is present and 0 if it was removed, β_1_ is the corresponding effect size, *X*_*i*2_ is a binary variable taking value 1 if the wheat type is soft and 0 hard, β_2_ is the corresponding effect size, *G*_*ij*_ is a dummy variable that encodes genotype (i.e., if the *i*th observation was taken on a plant belonging to the *j*th hybrid then *G*_*ij*_ = 1 and $G_{ij^{\prime }}=0$ for all *j*′≠*j*), α_*j*_ is the corresponding effect size, and ϵ_*i*_ is the error term. To avoid identifiability issues, the dummy variables encoding genotype were constructed with respect to a chosen baseline. It is important to note that through the aforementioned specifications model (5) roughly stratifies the genotypes based on a common phenotype, while model (6) further refines the analysis. An Analysis of Variance (ANOVA) was then conducted to determine effect of the leaf sheath on stalk flexural stiffness and stalk bending strength. This was accomplished using the *anova* function in R.

### Statistical Analyses: Relationship Between Flexural Rigidity and Bending Strength

A series of multiple linear regression models were fit to the transformed data to quantify the relationship between flexural rigidity and bending strength. These models take the following form:

(7) $$\begin{array}{r} Y_{i}=\beta_{0}+\beta_{1}X_{i1}+\beta_{2}Z_{i}+{\beta_{3}X}_{i2}+\epsilon_{i} \end{array}$$

(8) $$\begin{array}{r} Y_{i}=\beta_{0}+\beta_{1}X_{i1}+\beta_{2}Z_{i}+\sum \alpha_{j}G_{ij}+\epsilon_{i} \end{array}$$

where *Y*_*i*_ denotes the square root of bending strength, *Z*_*i*_ is the square root of flexural rigidity, and all other variables retain their previous definition.

### Statistical Analyses: Quantifying the Effect of the Leaf Sheath on Failure Patterns

The influence of the leaf sheath on the failure mode of the wheat was investigated. Three failure modes were considered; namely crushed, splintered, and snapped. To analyze these data, we fit a multinomial logit regression model with sheath presence and wheat type as the only predictor variables with both being entered into the model in an additive fashion. A multinomial logit regression model extends the common logistic regression model to a multi-category outcome (e.g., the outcome could be snapped, crushed, or splintered). As a part of this process, the model attempts to quantify K category specific probabilities, which leads to the use of K-1 linear predictors. Given that we have three categories we estimate as a part of one model 2 linear predictors, each having an intercept. In the results presented below, the outcome snapped is treated as the baseline category, and used to determine the odds of being crushed vs. snapped and the odds of being splintered vs. snapped. A second analysis is then conducted using crushed as a baseline category to determine if the sheath presence increases or decreases the odds of being crushed vs. splintered. For further details on multinomial logit regression models see Bilder and Loughin (2014).

## Results

### Effect of the Leaf Sheath on Bending Strength and Flexural Rigidity

**Figure 3:**
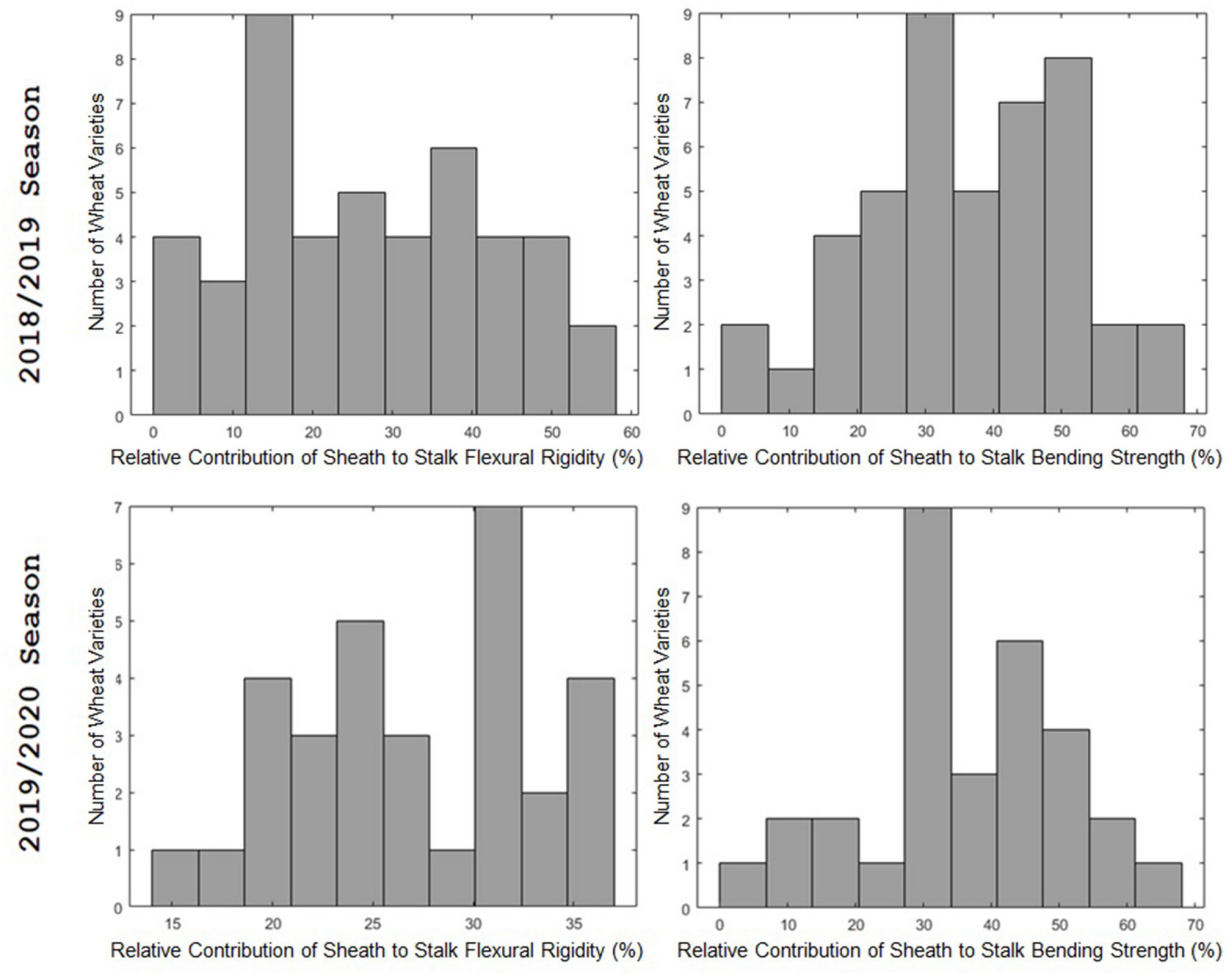
A histogram of the relative effect of the leaf sheath on stalk flexural rigidity (left) and stalk bending strength (right). The 2018/2019 season is shown in the top row; the 2019/2020 season is shown in the bottom row. The 2018/2019 season includes data from 45 varieties. The 2019/2020 season includes data from 31 varieties. The relative effect of the leaf sheath on stalk bending strength and stalk flexural rigidity varied from variety to variety. For some varieties the leaf sheath accounted for more than 60% of stalk bending strength and more than 50% of stalk flexural rigidity. In other words, in some cases the leaf sheath was more effective in resisting stalk lodging than the actual stem. However, for other varieties the contribution of the leaf sheath to flexural rigidity and bending strength was <10%.

**Figure 4:**
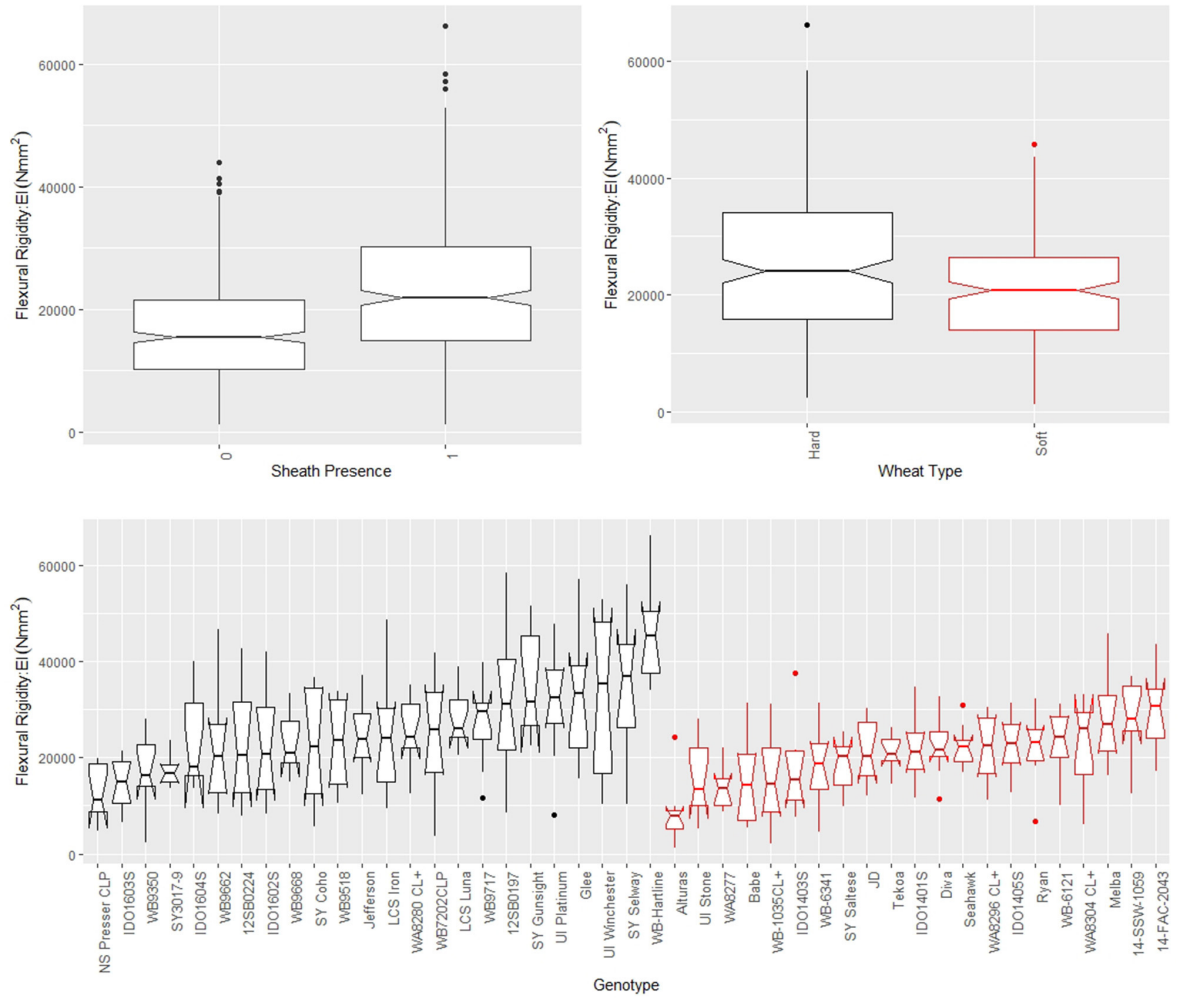
Boxplots of bending strength stratified by sheath presence (top left), wheat type (top right) and genotype (bottom) for data from the 2018/2019 season, where genotype is based on non-replicated plots. Data from the 2019/2020 season is presented in the Supplementary Material. In the top left panel, a sheath presence of 0 indicates the sheath was removed (i.e., stem strength) whereas a sheath presence of 1 indicates the sheath was not removed (i.e., stalk strength). In the top right and bottom panels soft wheat is shown in red and hard wheat is shown in black.

**Figure 5:**
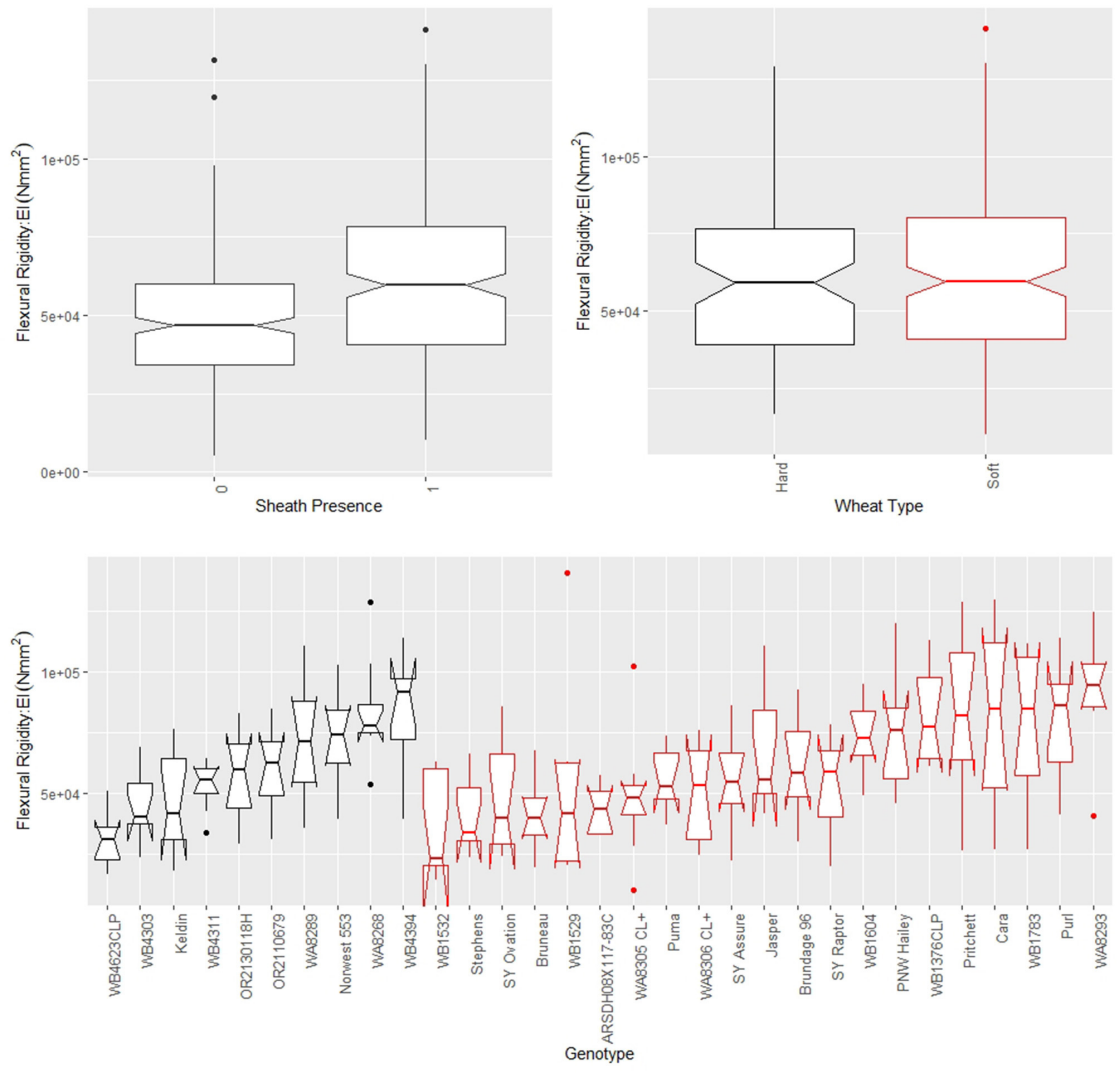
Boxplots of flexural rigidity stratified by sheath presence (top left), wheat type (top right) and genotype (bottom) for data from the 2018/2019 season, where genotype is based on non-replicated plots. Data from the 2019/2020 season is presented in the Supplementary Material. In the top left panel, a sheath presence of 0 indicates the sheath was removed (i.e., stem flexural rigidity) whereas a sheath presence of 1 indicates the sheath was not removed (i.e., stalk flexural rigidity). In the top right and bottom panels soft wheat is shown in red and hard wheat is shown in black.

The leaf sheath was found to have a large effect on the stalk flexural rigidity and the stalk bending strength of wheat. At the plot level, the sheath increased the flexural rigidity of the wheat stalks an average of 27.6% (+/– 15.4% standard deviation) and increased the bending strength by 36.7% (+/– 15.4% standard deviation) for the 2018/2019 season. For the 2019/2020 season the leaf sheath increased stalk flexural rigidity by an average of 26.9% (+/– 6.1% standard deviation) and increased stalk bending strength by an average of 35.6% (+/– 14.6% standard deviation). Analysis of paired data in which the same specimens were tested both with and without a leaf sheath indicated the leaf sheath accounted for 41.5% (2018/2019) and 27.8% (2019/2020) of the flexural rigidity of the stalk. Figure 3 depicts a histogram of the average relative contribution of the leaf sheath to stalk flexural stiffness and stalk bending strength for all genotypes (i.e., plots) measured in both the 2018/2019 and 2019/2020 seasons. Figures 4, 5 provide a depiction of the variation (via boxplots) in bending strength and flexural rigidity stratified by sheath presence, wheat type (hard or soft), and by genotype (i.e., plot) for the 2018/2019 season (see Supplementary Material for 2019/2020 season data). Both bending strength and flexural rigidity vary across these measures, with stronger associations being tied to sheath presence and genotype.

From models (5) and (6), we find that sheath presence is highly associated with the square root of flexural rigidity. These findings are summarized in Table 1 which presents the results of the ANOVA for flexural rigidity. The presence of the sheath is estimated to increase the mean of the square root of flexural rigidity by $\sim 23.69\;\sqrt{Nmm^{2}}$ (*p*-value <2e-16) (2018/2019 season), and $27.87\;\sqrt{Nmm^{2}}$ (*p*-value 1.7e-9) (2019/2020 season). Similarly, we find that sheath presence is also highly associated with the square root of bending strength. That is, based on these results we estimate that the presence of the sheath increases the mean of the square root of bending strength by $\sim 5.48\;\sqrt{Nmm}$ (*p*-value <2e-16) (2018/2019 season), and $5.31\;\sqrt{Nmm}$ (*p*-value <2.2e-16) (2019/2020 season). Although, it is worth noting that under model (5) we do find that genotype is a significant predictor (*p*-value <2e-16 [both 2018/2019 and 2019/2020 season]). These findings are summarized in Table 2 which presents the ANOVA results for stalk bending strength.

**Table 1:** ANOVA results of flexural rigidity, sheath presence, and wheat type (Model 4); ANOVA results of flexural rigidity, sheath presence, and genotype (Model 5).

**Model**	**$\sqrt{\text{F}lexural\;Rigidity\;}$**	**Df**	**Sum Sq**.	**Mean Sq**.	***F*-value**	***P*-value**
(4)	Sheath Presence	1	110868	110868	88.5	<2.2e-16
	Wheat Type	1	21558	21558	17.2	3.7e-05
	Residual	788	986963	1252		
(5)	Sheath Presence	1	110868	110868	108.4	<2.2e-16
	Genotype	44	246690	5607	5.5	<2.2e-16
	Residual	745	761831	1023		

**Table 2:** ANOVA results of bending strength, sheath presence, and wheat type (Model 4); ANOVA results of bending strength, sheath presence, and genotype (Model 5).

**Model**	**$\sqrt{\text{B}ending\;Strengt\text{h}}$**	**Df**	**Sum Sq**.	**Mean Sq**.	***F*-value**	***P*-value**
(4)	Sheath Presence	1	5930.0	5930.0	210.9	<2.2e-16
	Wheat Type	1	44.7	44.7	1.6	0.2
	Residual	788	22153.4	28.1		
(5)	Sheath Presence	1	5930.0	5930.0	249.0	<2.2e-16
	Genotype	44	4458.7	101.3	4.3	<2.2e-16
	Residual	745	17739.4	23.8		

### Relationship Between Flexural Rigidity and Bending Strength

**Figure 6:**
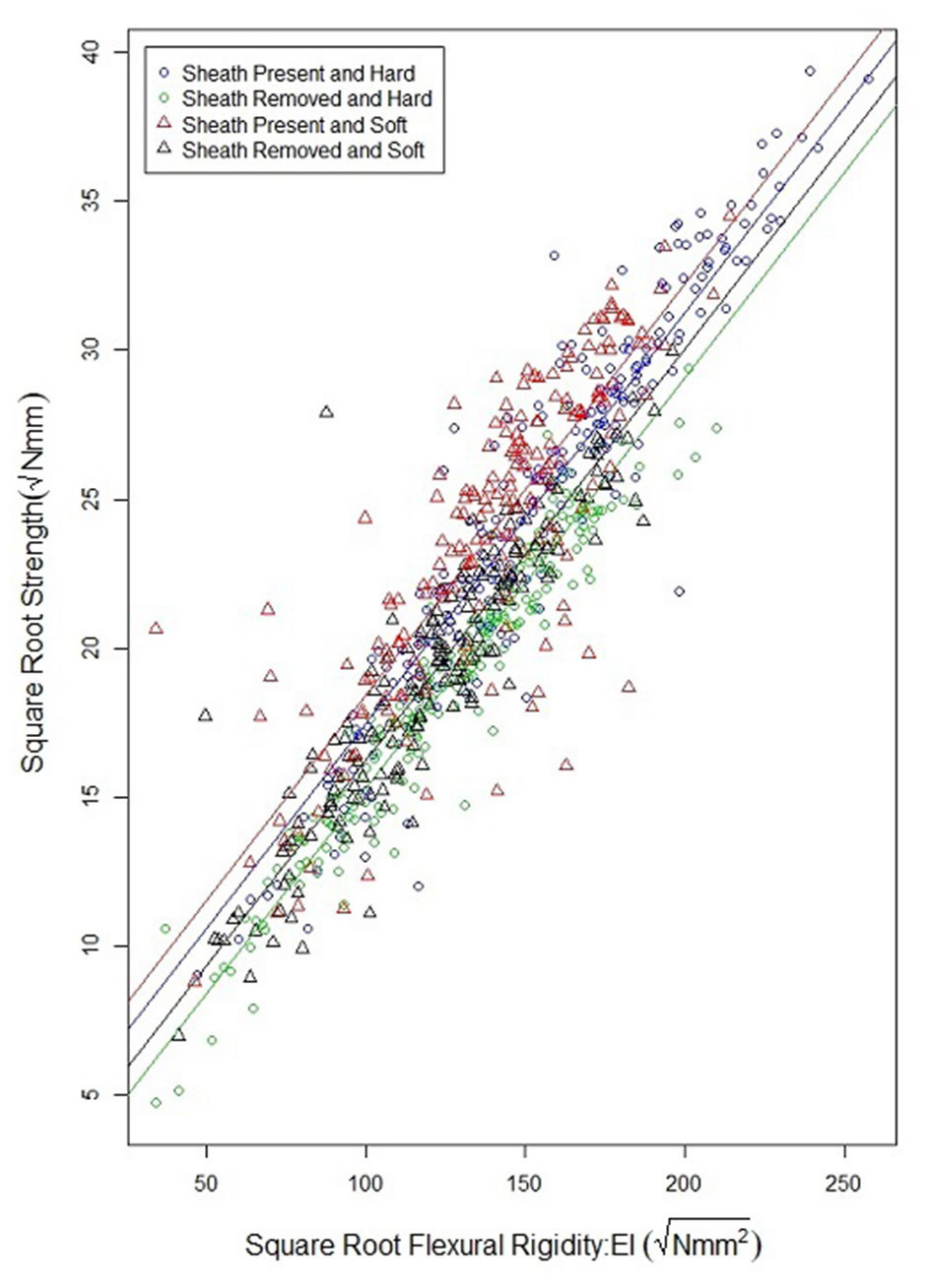
Scatterplot of the square root of bending strength and the square root of flexural rigidity for hard and soft wheat, both with and without a leaf sheath. Flexural rigidity and bending strength were strongly correlated as has been shown in previous studies. Moreover, the relationship between flexural rigidity and bending strength is relatively independent of wheat type, or sheath presence.

As observed in previous studies a strong relationship exists between bending strength and flexural rigidity (Robertson et al., 2016; Stubbs et al., 2018, 2019b). Figure 6 provides a scatter plot relating flexural rigidity and bending strength while stratifying the data by sheath presence and wheat type. Table 3 summarizes the ANOVA results which compare bending strength, flexural rigidity, sheath presence, wheat type and genotype. The multiple R-squared values for models (7) and (8) are 0.87 and 0.90, respectively (2018/2019 season), and 0.70 and 0.87, respectively (2019/2020 season).The R-squared value for the reduced model which considers only flexural rigidity was 0.83 (2018/2019 season) and 0.61 (2019/2020 season). This finding suggests that most of the variation in bending strength can be explained by flexural rigidity. It is important to note that the slope of all regression lines in Figure 6 are very similar. These results imply that the reduction in flexural rigidity due to removal of the leaf sheath is a good estimate of the reduction in bending strength due to removal of the leaf sheath.

**Table 3:** ANOVA results of bending strength, flexural rigidity, sheath presence, and wheat type (Model 6); ANOVA analysis of bending strength, flexural rigidity, sheath presence, and genotype (Model 7).

**Model**	**$\sqrt{\text{S}trengt\text{h}}$**	**Df**	**Sum Sq**.	**Mean Sq**.	***F*-value**	***P*-value**
(6)	$\sqrt{Flexural\;Rigidity\;}$	1	23479.0	23479.0	5202.3	<2.2e-16
	Sheath Presence	1	919.6	919.6	203.8	<2.2e-16
	Wheat Type	1	177.6	177.6	39.4	5.8e-10
	Residual	787	3551.9	4.5		
(7)	$\sqrt{Flexural\;Rigidity\;}$	1	23479.0	23479.0	5961.2	<2.2e-16
	Sheath Presence	1	919.6	919.6	233.5	<2.2e-16
	Genotype	44	799.2	18.2	4.6	<2.2e-16
	Residual	744	2930.3	3.9		

### Effect of the Leaf Sheath on Failure Type

The leaf sheath had a significant effect on the failure mode of the tested samples. For samples tested with the leaf sheath intact 66.8% crushed, 21.9% snapped and 11.3% splintered. For samples tested with the leaf sheath removed 27.7% crushed, 71.7% snapped and 0.5% splintered. A multinomial logit regression was conducted to quantify statistical differences in failure mode due to the presence or absence of the leaf sheath. This analysis revealed that the presence of the leaf sheath increases the odds of being crushed vs. snapped by 12.4 (2018/2019 season) and 3.46 (2019/2020 season). Similarly, the presence of the sheath increases the odds of being splintered vs. snapped by 18.0 (2018/2019 season) and 5.75 (2019/2020 season). These findings are summarized in Table 4. A second multinomial logit regression revealed that the presence of the leaf sheath did not increase the odds of being crushed vs. splintered (results table not shown). In other words when the leaf sheath is intact the stalk is more likely to crush or splinter and less likely to snap. Moreover, wheat type was found to be insignificant.

**Table 4:** Multinomial logit regression of failure type, sheath presence, and wheat type.

**Failure mode**	**Predictor**	**Estimate**	**Std. Error**	***P*-value**
Crushed	Intercept	−1.15	0.19	9.0e-10
	Sheath Presence	2.52	0.24	<2.2e-16
	Wheat Type	−0.34	0.25	0.17
Splintered	Intercept	−2.76	0.34	6.6e-16
	Sheath Presence	2.89	0.37	9.5e-10
	Wheat Type	0.15	0.33	0.65
				

### Effect of the Sheath on Moment of Inertia and Flexural Rigidity

**Figure 7:**
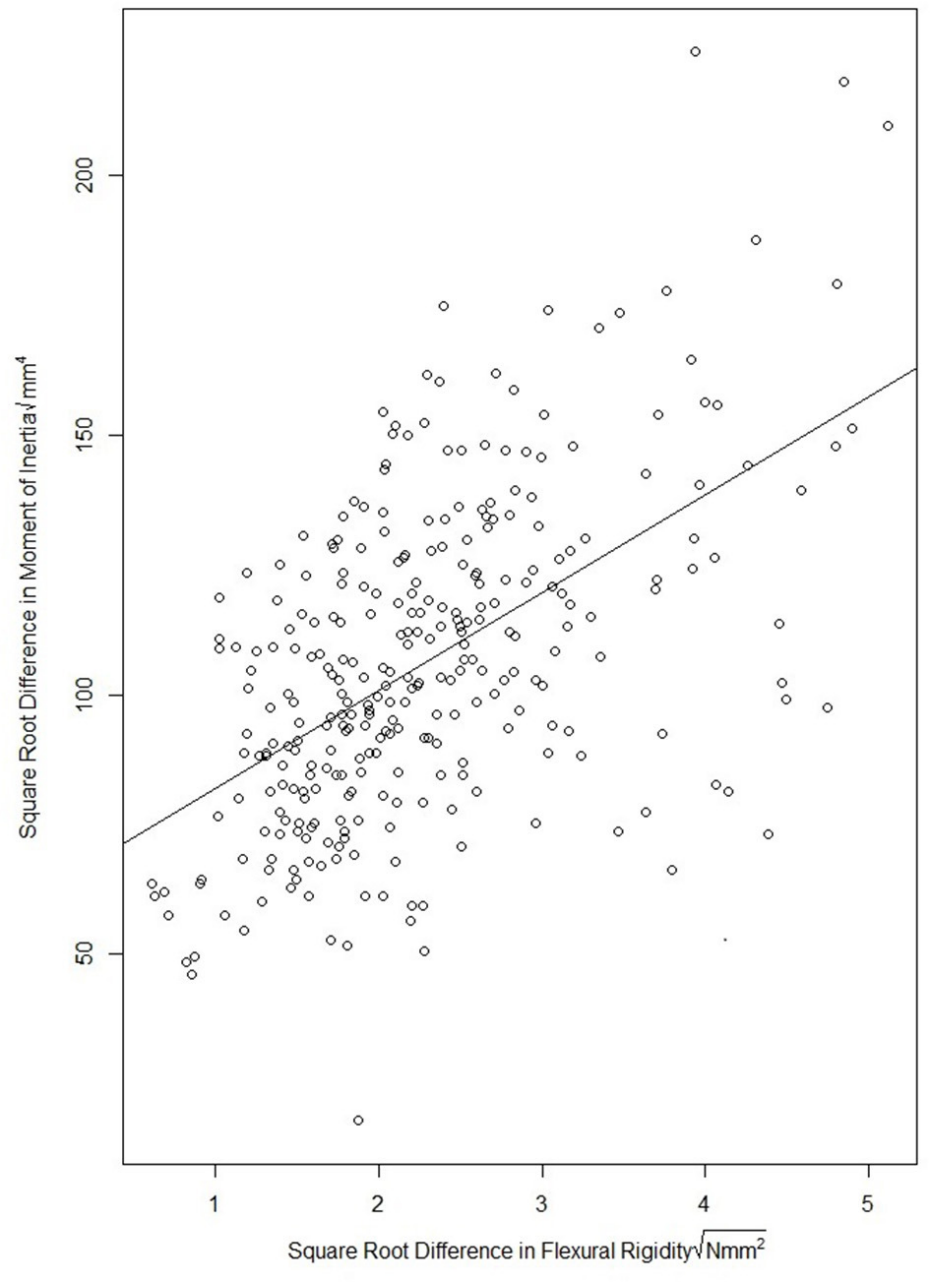
Scatter plot of the square root difference in flexural rigidity due to removal of the leaf sheath vs. the square root difference in moment of inertia due to removal of the leaf sheath. The figure illustrates that the change in moment of inertia due to removal of the leaf sheath does not fully account for the reduction in flexural rigidity of the stem. This suggests more complex interactions between the leaf sheath and stem are at play that influence stalk flexural rigidity.

The effect of the leaf sheath on the moment of inertia and flexural rigidity of stalk specimen was investigated through a paired data analysis. In particular, the moment of inertia and flexural rigidity of each stalk specimen were measured both before and after the sheath was removed. Figure 7 provides a scatterplot of difference in flexural rigidity and the difference in moment of inertia of each stalk due to removal of the leaf sheath. To formally quantify the association, a simple linear regression model was fit using the square root of the difference in flexural rigidity as the dependent variable and square root of the difference in moment of inertia as the independent variable. This analysis revealed a significant association (*p*-value <2.2e-16, both 2018/2019 season and 2019/2020 season) and resulted in a fitted model with an R-squared of 0.29 (both 2018/2019 and 2019/2020 season). The fairly poor association between flexural rigidity and moment of inertia in this analysis suggests that the reinforcing role of the leaf sheath may be complex. In other words, the leaf sheath may not simply increase the flexural rigidity and bending strength of the stem by merely increasing the moment of inertia.

## Discussion

The reinforcing role of the clasping leaf sheath in grass species was first investigated by Karl Niklas in the 1990's (Niklas, 1990, 1998). In his studies, Niklas estimated that the leaf sheath could increase the bending stiffness and strength of grasses by nearly 50%. Despite these remarkable findings only a single case study has been published (which the authors are aware of) investigating the role of the leaf sheath in stalk lodging events for wheat (Wu and Ma, 2019). Results of our study demonstrate the leaf sheath plays an essential role in resisting forces that induce wheat stalk lodging. For some of the wheat plots included in our study the leaf sheath had a greater effect on bending strength than the stem itself.

### Comparison of Results to Previously Published Data

The only previous study directly investigating the leaf sheath of wheat stems was a *case study* that analyzed two cultivars grown in a single environment 10 days prior to maturity (Wu and Ma, 2019). Our study looked at over 70 cultivars and analyzed the plants at the time of harvest. Both studies demonstrated the leaf sheath significantly increased the bending strength and flexural rigidity of wheat stalks. Given the broad sampling design implemented in our study it is reasonable to assume as a general principle that the leaf sheath has a large effect on stalk biomechanics and significantly reinforces the stem of wheat plants to resist stalk lodging. Wu and Ma (2019) highlighted that it is generally believed that when plants approach physiological maturity and the leaf sheath senesces its biomechanical role is greatly diminished. However, data from our study demonstrates that even at harvest time (after the plant has senesced) the leaf sheath still plays a critical biomechanical role. In several of the plots included in this study the relative contribution of the leaf sheath to stalk bending strength and stalk flexural rigidity was >50%. In other words, even in fully mature (senesced) plants the biomechanical role of the leaf sheath is sometimes greater than the biomechanical role of the stem itself. More studies are needed to fully determine how the biomechanical role of the leaf sheath changes throughout the plant's entire lifecycle. However, qualitative field observations from our lab as well as data published in the literature suggest the biomechanical role of the leaf sheath to be even greater in young plants than it is in fully mature plants (Wu and Ma, 2019).

The complex biomechanical mechanisms through which the leaf sheath increases the bending strength and flexural rigidity of wheat stalks remains to be fully elucidated. Data collected in the case study by Wu and Ma (2019) indicated that the sheath increases the bending strength and flexural rigidity of wheat stalks by increasing their moment of inertia. However, the wider range of cultivars utilized in our study demonstrated this is not generally the case. In our study the increase in the moment of inertia due to the presence of the leaf sheath was poorly correlated with the increase in flexural rigidity due to the presence of the sheath (*R*^2^ = 0.29). This indicates that other complex interactions between the leaf sheath and stem are likely at play. This notion is supported by observations that the stem typically breaks at the moment it becomes separated from the leaf sheath and that the leaf sheath unravels prior to the stem breaking. In other words, the influence of the sheath on the bending strength and flexural rigidity of the stalk appears to be more complex than the simple addition of structural material. The authors hypothesize that other factors, such as the friction between the sheath and stem, the unwrapping of the sheath during the bending of the stem, and the radial preload of the sheath acting upon the stem influence the interaction between the leaf sheath and stem, and therefore also influence the bending strength of the stalk. This notion is further supported by results of the failure patterns analysis (there is a significant change in failure pattern when the leaf sheath is removed). Further research into the identification and quantification of complex interactions that may exist between the stem and sheath are key to fully understanding the biomechanical role of the leaf sheath. In addition, further studies are needed to identify the most influential physical characteristics of the leaf sheath (e.g., thickness, stiffness, degree of wrapping, etc.).

### Implications for Breeding

The contribution of the leaf sheath to stalk lodging resistance may be genotype dependent. Some plots in this study saw large reductions in bending strength and flexural rigidity with removal of the leaf sheath whereas others experienced relatively smaller reductions. However, genotypes were not replicated in this study. Further research is therefore required to determine if the biomechanical characteristics of the leaf sheath are driven by genetic factors (G), environmental factors (E), or the interaction between them (G x E). Identifying genetic and environmental features that affect sheath biomechanics (e.g., sheath thickness, stiffness, strength, degree of wrapping etc.) is a promising avenue to increase stalk lodging resistance in wheat. The biomass of the leaf sheath is significantly less than the biomass of the stem. Modifying the leaf sheath through selective breeding may therefore have a lesser effect on yield as compared to modifying the stem. However, a phenotyping protocol must first be established to enable selective breeding and functional genomic studies of the biomechanical role of the leaf sheath.

### Phenotyping Protocol

Flexural rigidity is a reasonably accurate proxy measurement for stalk bending strength. The flexural rigidity of wheat stalks can be measured through non-destructive bending tests conducted with and without leaf sheaths. Moreover, the relationship between flexural rigidity and stalk bending strength is not significantly affected by the presence or lack of the leaf sheath (see regression lines in Figure 6). Therefore, it is possible to reasonably estimate the relative contribution of the leaf sheath toward stalk bending strength by determining the relative contribution of the leaf sheath toward stalk flexural rigidity. In particular, the following phenotyping protocol could be used by future studies to investigate the genetic underpinnings of the biomechanical role of the leaf sheath: (1) harvest the wheat stalk by cutting the plant at the ground and just below the panicle, (2) test the stalk in three-point bending—ensuring an aspect ratio of at least 10:1—within the elastic range of the material. The stalk should be loaded and unloaded 10 times as detailed in the methods section of this paper. This is done to ensure the test fixture is fully seated on the stalk sample and to minimize viscoelastic effects. (3) carefully remove the leaf sheath while ensuring no damage has occurred to the stem, (4) re-test the stem without the leaf sheath for 10 cycles using the same three-point bending test setup as before, (5) the difference in flexural rigidity of the sample with and without the leaf sheath is a reasonable estimate of the difference in stalk bending strength. It should be noted that several field-based phenotyping devices capable of measuring flexural rigidity have been developed and that these devices could be used in place of the universal testing system utilized in the current study (Cook et al., 2019; Heuschele et al., 2019; Erndwein et al., 2020). However, if field-based phenotyping methods are employed the grain head should either be removed prior to testing or alternatively the grain head should be weighed and the effect of the grain weight on bending strength and flexural rigidity measurements should be accounted for as outlined in Stubbs et al. (2020a). In addition, when conducting field tests, it is important to either remove adjacent plants to prevent plant to plant interactions or to account for interactions among plants through a mathematical model (Bebee et al., 2021).

While the phenotyping protocol outlined above is feasible, it is time consuming. Future studies should seek to develop higher throughput phenotyping methodologies capable of measuring wheat stalk flexural stiffness and stalk bending strength. Furthermore, high throughput phenotyping methods capable of rapidly characterizing the biomechanical features of the leaf sheath are needed.

### Limitations

Stalk samples used in this study were fully mature and were free of disease and pest damage. Diseased or pest damaged stalks were not included to limit extraneous variables. In addition, this study was performed on two years' worth of specimens, but the genotypes were not replicated, hence the authors were unable to conclusively differentiate genetic and environmental effects in this study. This study does not aim to make any conclusions on specific cultivar/genotype effects of the leaf sheath. Instead, this study attempts to highlight a phenotype that has been historically overlooked when making breeding decisions, and present a path forward for simple, phenotyping strategies. This study analyzed only the most basal portion of wheat stalks. This was done to enable a more direct comparison between different wheat varieties. However, future work should investigate more apical sections of the plant as well.

In this study the leaf sheath is accounted for in an admittedly crude manner. For example, in measuring geometry, simple caliper measurements of the radii were taken where optical analysis or computed tomography would give more precise measurements (e.g., Seegmiller et al., 2020). In addition, Equations 3 and 4 assume that (1) the sheath only wraps around the stem a single time, (2) the amount of wrap is consistent, and (3) the leaf sheath has a constant thickness. More detailed modeling of the geometry of the sheath and stem will be required to investigate the limitations of these assumptions and to develop deeper mechanistic understanding. A host of future studies will likely be required to fully understand the biomechanical role of the clasping leaf sheath and its genetic underpinnings. In particular, future studies are needed to investigate the tissue stiffness, tissue strength and thickness of the leaf sheath. In addition, it is currently unclear how the biomechanical contribution of the leaf sheath changes throughout the life cycle of the plant. Future experimental and computational modeling studies may be needed to determine why removal of the leaf sheath causes the failure pattern of the stalk to change. Research in this area is expected to ultimately improve the stalk lodging resistance of future wheat varieties.

## Conclusion

The clasping leaf sheath plays an integral role in determining the bending strength and failure mode of wheat. Results demonstrated that in some cases the leaf sheath can have a bigger effect on stalk bending strength than the stem itself. However, the complex biomechanical role of the clasping leaf sheath has yet to fully elucidated and many future studies will be required to generate needed understanding in this area. Research in this area is expected to improve the stalk lodging resistance of wheat. In general, future studies on the topic of wheat stalk lodging may benefit from analyzing the leaf sheath and stem as two separate and distinct structures. Most previous studies have either focused solely on the stem or on the combined sheath-stem complex. Separating these two structures will enable greater specificity and therefore improve the outcome of ‘omics' investigations of wheat stalk lodging.

## Data Availability Statement

The raw data supporting the conclusions of this article will be made available by the authors, without undue reservation.

## Author Contributions

All authors were fully involved in the study and preparation of the manuscript. All authors contributed to the article and approved the submitted version.

## Conflict of Interest

The authors declare that the research was conducted in the absence of any commercial or financial relationships that could be construed as a potential conflict of interest. The reviewer DC declared a collaboration with several of the authors DR, CS to the handling editor.

## Publisher's Note

All claims expressed in this article are solely those of the authors and do not necessarily represent those of their affiliated organizations, or those of the publisher, the editors and the reviewers. Any product that may be evaluated in this article, or claim that may be made by its manufacturer, is not guaranteed or endorsed by the publisher.

## References

[B1] Al-Zube L. Sun W. Robertson D. Cook D. (2018). The elastic modulus for maize stems. Plant Methods. 14:11. 10.1186/s13007-018-0279-6 29449871 PMC5806466

[B2] Bashford L. L. Maranville J. W. Weeks S. A. Campbell R. (1976). Mechanical-properties affecting lodging of sorghum. Trans. ASAE 19, 962–966. 10.13031/2013.36155

[B3] Bebee A. Stubbs C. J. Robertson D. J. (2021). Large deflection model for multiple, inline, interacting cantilever beams. J. Appl. Mech. 88:041005. 10.1115/1.4049072

[B4] Beer F. P. Russell Johnston E. DeWolf J. T. Mazurek D. F. (2012). Mechanics of Materials, 6th Edn. New York, NY: Mc Graw Hill.

[B5] Berry P. Sylvester-Bradley R. Berry S. (2007). Ideotype design for lodging-resistant wheat. Euphytica 154, 165–179. 10.1007/s10681-006-9284-3

[B6] Berry P. M. Spink J. (2012). Predicting yield losses caused by lodging in wheat. Field Crops Res 137, 19–26. 10.1016/j.fcr.2012.07.019

[B7] Berry P. M. Spink J. Sterling M. Pickett A. A. (2003b). Methods for rapidly measuring the lodging resistance of wheat cultivars. J. Agron. Crop Sci. 189, 390–401. 10.1046/j.0931-2250.2003.00062.x

[B8] Berry P. M. Spink J. H. Gay A. P. Craigon J. (2003a). A comparison of root and stem lodging risks among winter wheat cultivars. J. Agric. Sci. 141, 191–202. 10.1017/S002185960300354X

[B9] Berry P. M. Sterling M. Spink J. H. Baker C. J. Sylvester-Bradley R. Mooney S. J.. (2004). “Understanding and reducing lodging in cereals,” in Advances in Agronomy, ed D. L. Sparks (Cambridge, MA: Academic Press), 217–271. 10.1016/S0065-2113(04)84005-7

[B10] Bilder C. R. Loughin T. M. (2014). Analysis of Categorical Data With R. Boca Raton, FL: CRC Press. 10.1201/b17211

[B11] Cook D. D. de la Chapelle W. Lin T. C. Lee S. Y. Sun W. Robertson D. J.. (2019). DARLING: a device for assessing resistance to lodging in grain crops. Plant Methods. 15:102. 10.1186/s13007-019-0488-7 31497063 PMC6720399

[B12] Daamen R. A. Wijnands F. G. Vliet G. vander. (1989). Epidemics of diseases and pests of winter wheat at different levels of agrochemical input: a study on the possibilities for designing an integrated cropping system. J. Phytopathol. 125, 305–319. 10.1111/j.1439-0434.1989.tb01075.x

[B13] Easson D. White E. Pickles S. (1993). The effects of weather, seed rate and cultivar on lodging and yield in winter wheat. J. Agric. Sci. 121, 145–156. 10.1017/S0021859600077005

[B14] Erndwein L. Cook D. D. Robertson D. J. Sparks E. E. (2020) Field-based mechanical phenotyping of cereal crops to assess lodging resistance. Appl. Plant Sci. 8:e11382. 10.1002/aps3.11382 32995102 PMC7507486

[B15] Heuschele D. J. Wiersma J. Reynolds L. Mangin A. Lawley Y. Marchetto P. (2019). The Stalker: An open source force meter for rapid stalk strength phenotyping. HardwareX. 6:e00067. 10.1016/j.ohx.2019.e00067

[B16] Keller M. Karutz C. Schmid J. E. Stamp P. Winzeler M. Keller B.. (1999). Quantitative trait loci for lodging resistance in a segregating wheat × spelt population. Theor. Appl. Genet. 98, 1171–1182. 10.1007/s001220051182

[B17] Kong E. Liu D. Guo X. Yang W. Sun J. Li X.. (2013). Anatomical and chemical characteristics associated with lodging resistance in wheat. Crop J. 1, 43–49. 10.1016/j.cj.2013.07.012

[B18] Loyce C. Meynard J. M. Bouchard C. Rolland B. Lonnet P. Bataillon P.. (2008). Interaction between cultivar and crop management effects on winter wheat diseases, lodging, and yield. Crop Prot. 27, 1131–1142. 10.1016/j.cropro.2008.02.001

[B19] Mäkinen H. Kaseva J. Trnka M. Balek J. Kersebaum K. C. Nendel C.. (2018). Sensitivity of European wheat to extreme weather. Field Crops Res. 222, 209–217. 10.1016/j.fcr.2017.11.008

[B20] Niklas K. J. (1990). The mechanical significance of clasping leaf sheaths in grasses: evidence from two cultivars of *Avena sativa*. Ann. Bot. 65, 505–512. 10.1093/oxfordjournals.aob.a087962

[B21] Niklas K. J. (1998). The mechanical roles of clasping leaf sheaths: evidence from *Arundinaria tecta* (Poaceae) shoots subjected to bending and twisting forces. Ann. Bot. 81, 23–34. 10.1006/anbo.1997.0513

[B22] Robertson D. Smith S. Gardunia B. Cook D. (2014). An improved method for accurate phenotyping of corn stalk strength. Crop Sci. 54:2038. 10.2135/cropsci2013.11.0794

[B23] Robertson D. J. Julias M. Gardunia B. W. Barten T. Cook D. D. (2015a). Corn stalk lodging: a forensic engineering approach provides insights into failure patterns and mechanisms. Crop Sci. 55:2833. 10.2135/cropsci2015.01.0010

[B24] Robertson D. J. Julias M. Lee S. Y. Cook D. D. (2017). Maize stalk lodging: morphological determinants of stalk strength. Crop Sci. 57:926. 10.2135/cropsci2016.07.0569

[B25] Robertson D. J. Lee S. Y. Julias M. Cook D. D. (2016). Maize stalk lodging: flexural stiffness predicts strength. Crop Sci. 56:1711. 10.2135/cropsci2015.11.0665

[B26] Robertson D. J. Smith S. L. Cook D. D. (2015b). On measuring the bending strength of septate grass stems. Am. J. Bot. 102, 5–11. 10.3732/ajb.1400183 25587143

[B27] Schulgasser K. Witztum A. (1992). On the strength, stiffness and stability of tubular plant stems and leaves. J. Theor. Biol. 155, 497–515. 10.1016/S0022-5193(05)80632-0

[B28] Seegmiller W. Graves J. Robertson D. J. (2020). A novel rind puncture technique to measure rind thickness and diameter in plant stalks. Plant Methods. 16:44. 10.1186/s13007-020-00587-4 32266000 PMC7110687

[B29] Stubbs C. Baban N. Robertson D. Al-Zube L. Cook D. (2018). “Bending stress in plant stems: models and assumptions,” in Plant Biomechanics - From Structure to Function at Multiple Scales, eds A. Geitmann, J. Gril (Berlin: Springer Verlag), 49–77. 10.1007/978-3-319-79099-2_3

[B30] Stubbs C. J. Cook D. D. Niklas K. J. (2019a). A general review of the biomechanics of root anchorage. J. Exp. Bot. 70, 3439–3451. 10.1093/jxb/ery451 30698795

[B31] Stubbs C. J. Larson R. Cook D. D. (2019b). Maize stem buckling failure is dominated by morphological factors. bioRxiv [preprint]. 10.1101/833863

[B32] Stubbs C. J. Oduntan Y. A. Keep T. R. Noble S. D Robertson D. J. (2020a). The effect of plant weight on estimations of stalk lodging resistance. Plant Methods. 16:128. 10.1186/s13007-020-00670-w 32973914 PMC7507268

[B33] Stubbs C. J. Seegmiller K. McMahan C. Sekhon R. S. Robertson D. J. (2020b). Diverse maize hybrids are structurally inefficient at resisting wind induced bending forces that cause stalk lodging. Plant Methods 16, 1–15. 10.1186/s13007-020-00608-2 32426024 PMC7216590

[B34] Tripathi S. C. Sayre K. D. Kaul J. N. Narang R. S. (2002). Effect of planting methods and N rates on lodging, morphological characters of culm and yield in spring wheat varieties. Cereal Res. Commun. 30, 431–438. 10.1007/BF03543440

[B35] Tripathi S. C. Sayre K. D. Kaul J. N. Narang R. S. (2003). Growth and morphology of spring wheat (*Triticum aestivum* L.) culms and their association with lodging: effects of genotypes, N levels and ethephon. Field Crops Res. 84, 271–290. 10.1016/S0378-4290(03)00095-9

[B36] USDA (2013). World Agricultural Supply and Demand Estimates Report. United States Department of Agriculture.

[B37] van der Velde M. Tubiello F. N. Vrieling A. Bouraoui F. (2012). Impacts of extreme weather on wheat and maize in France: evaluating regional crop simulations against observed data. Clim. Change 113, 751–765. 10.1007/s10584-011-0368-2

[B38] Watson D. J. French S. A. W. (1971). Interference between rows and between plants within rows of a wheat crop, and its effects on growth and yield of differently-spaced rows. J. Appl. Ecol. 8:421. 10.2307/2402881

[B39] Wegst U. Ashby M. (2007). The structural efficiency of orthotropic stalks, stems and tubes. J. Mater. Sci. 42, 9005–9014. 10.1007/s10853-007-1936-8

[B40] Wu W. Ma B. (2019). The mechanical roles of the clasping leaf sheath in cereals: two case studies from oat and wheat plants. J. Agron. Crop Sci. 206, 118–129. 10.1111/jac.12362

[B41] Young W. C. Budynas R. G. (2002). Roark's Formulas for Stress and Strain. New York, NY: McGraw-Hill.

[B42] Zuber U. Winzeler H. Messmer M. M. Keller M. Keller B. Schmid J. E.. (1999). Morphological traits associated with lodging resistance of spring wheat (*Triticum aestivum* L.). *J. Agron. Crop Sci*. 182, 17–24. 10.1046/j.1439-037x.1999.00251.x

