# Supplementary material for: The Overlooked Biomechanical Role of the Clasping Leaf Sheath in Wheat Stalk Lodging
Source: Front Plant Sci. 2021 Aug 20;12:617880. doi: 10.3389/fpls.2021.617880 (PMC8417718; doi:10.3389/fpls.2021.617880)
Supplement: Supplementary file 1 [file Data_Sheet_1.docx]

**SUPPLEMENTARY DATA**

This supplementary data provides the statistical analysis figures and tables for the 2019/2020 season.

**
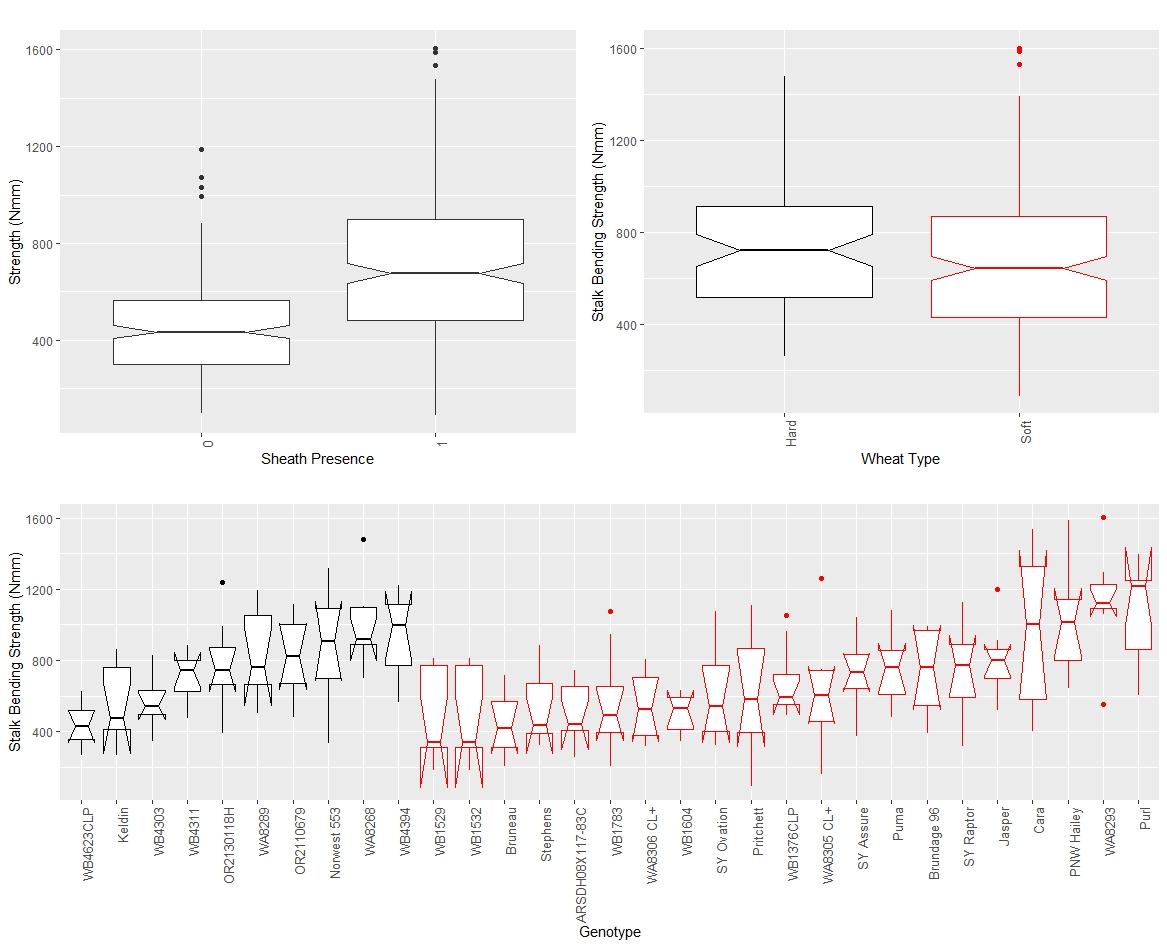
**

Figure S1: Boxplots of bending strength stratified by sheath presence (top left), wheat type (top right) and genotype (bottom) for data from the 2019/2020 season, where genotype is based on non-replicated plots. In the top left panel, a sheath presence of 0 indicates the sheath was removed (i.e., stem strength) whereas a sheath presence of 1 indicates the sheath was not removed (i.e., stalk strength). In the top right and bottom panels soft wheat is shown in red and hard wheat is shown in black.


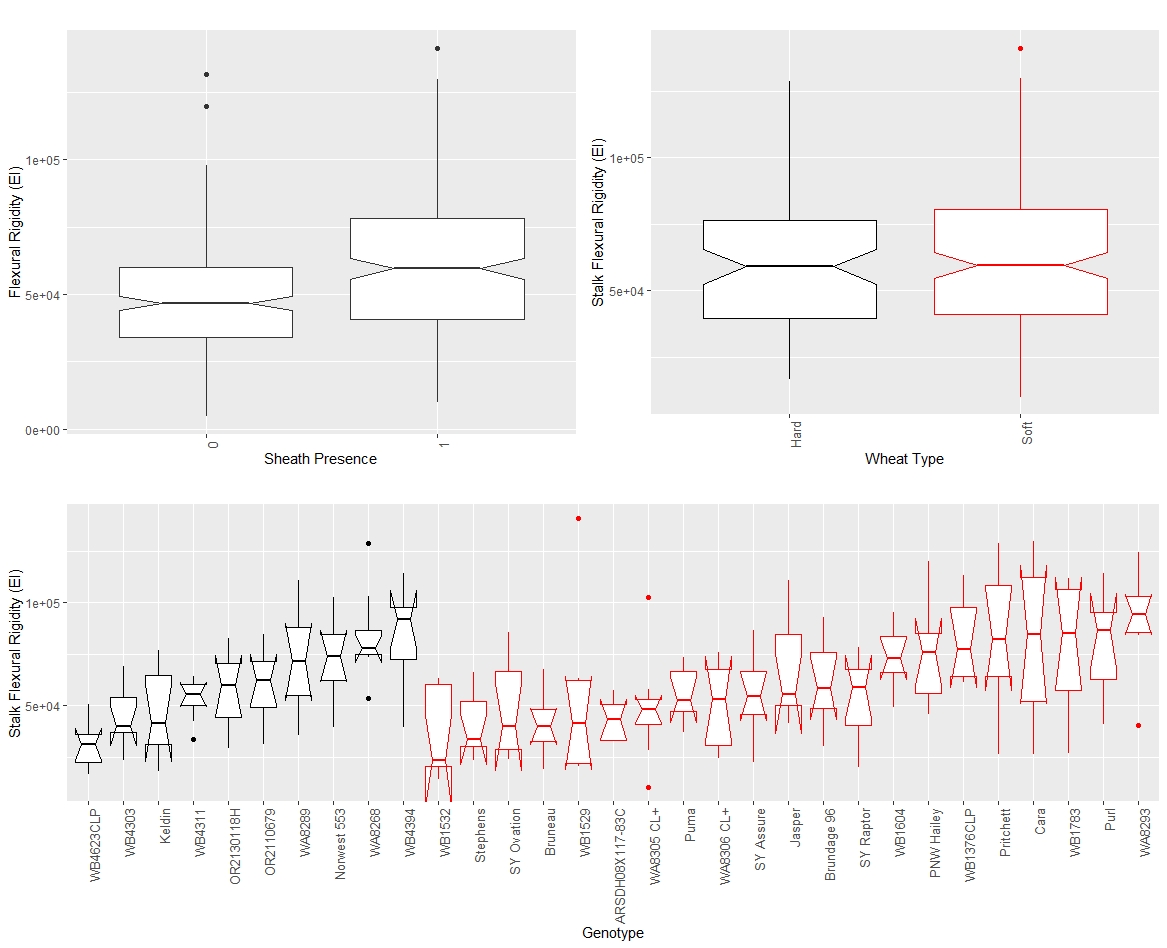


Figure S2: Boxplots of stalk flexural rigidity stratified by sheath presence (top left), wheat type (top right) and genotype (bottom) for data from the 2019/2020 season, where genotype is based on non-replicated plots. In the top left panel, a sheath presence of 0 indicates the sheath was removed (i.e., stem flexural rigidity) whereas a sheath presence of 1 indicates the sheath was not removed (i.e., stalk flexural rigidity). In the top right and bottom panels soft wheat is shown in red and hard wheat is shown in black.


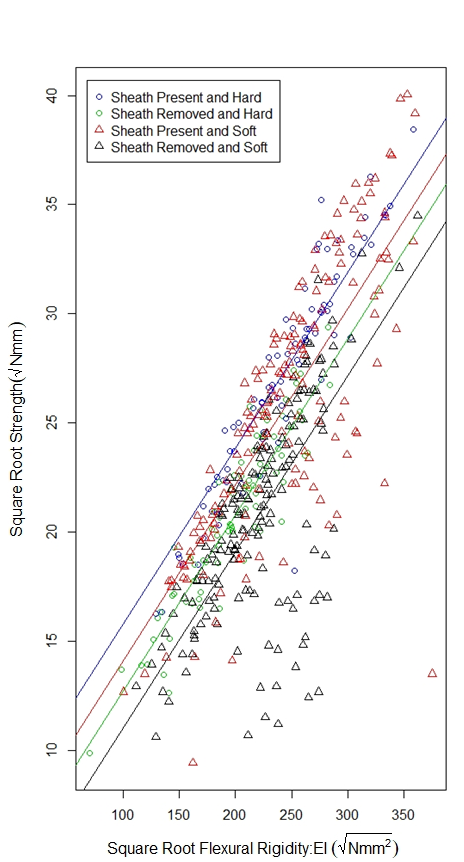


Figure S3: Scatterplot of the square root of stalk bending strength and the square root of stalk flexural rigidity for hard and soft wheat, both with and without a leaf sheath (data from 2019/2020 season). Stalk Flexural rigidity and stalk bending strength were strongly correlated as has been shown in previous studies. Moreover, the relationship between flexural rigidity and stalk bending strength is relatively independent of wheat type, or sheath presence.


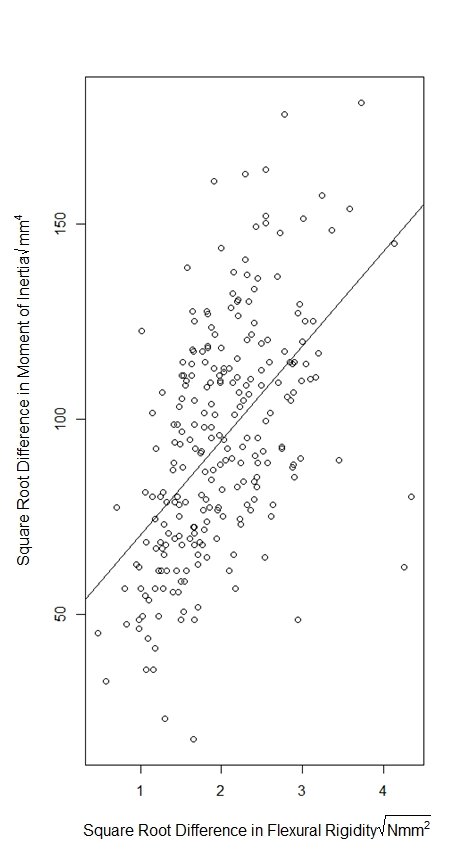


Figure S4: Scatter plot of the square root difference in flexural rigidity due to removal of the leaf sheath vs the square root difference in moment of inertia due to removal of the leaf sheath for data from 2019/2020 season. The figure illustrates that the change in moment of inertia due to removal of the leaf sheath does not fully account for the reduction in flexural rigidity of the stem. This suggests more complex interactions between the leaf sheath and stem are at play that influence stalk flexural rigidity.

**Tables**

*Table 1: ANOVA results of flexural rigidity, sheath presence, and wheat type (Model 4); ANOVA results of flexural rigidity, sheath presence, and genotype (Model 5)*

| Model | $\sqrt{Flexural Rigidity}$ | Df | Sum Sq. | Mean Sq. | F-value | P-value |
| --- | --- | --- | --- | --- | --- | --- |
| (4) | Sheath Presence | 1 | 96311 | 96311 | 37.7 | 1.7e-09 |
|  | Wheat Type | 1 | 14647 | 14647 | 5.7 | 0.017 |
|  | Residual | 493 | 1259458 | 2555 |  |  |
| (5) | Sheath Presence | 1 | 96311 | 96311 | 53.0 | 1.4e-12 |
|  | Genotype | 30 | 431367 | 14379 | 7.9 | <2.2e-16 |
|  | Residual | 464 | 842738 | 1816 |  |  |

*Table 2: ANOVA results of bending strength, sheath presence, and wheat type (Model 4); ANOVA results of bending strength, sheath presence, and genotype (Model 5)*

| Model | $\sqrt{Bending Strength}$ | Df | Sum Sq. | Mean Sq. | F-value | P-value |
| --- | --- | --- | --- | --- | --- | --- |
| (4) | Sheath Presence | 1 | 3499.3 | 3499.3 | 129.8 | <2.2e-16 |
|  | Wheat Type | 1 | 60.5 | 60.5 | 2.2 | 0.13 |
|  | Residual | 493 | 13289.9 | 27.0 |  |  |
| (5) | Sheath Presence | 1 | 3499.3 | 3499.3 | 188.3 | <2.2e-16 |
|  | Genotype | 30 | 4731.2 | 157.7 | 8.5 | <2.2e-16 |
|  | Residual | 464 | 8619.3 | 18.6 |  |  |

*Table 3: ANOVA results of bending strength, flexural rigidity, sheath presence, and wheat type (Model 6); ANOVA analysis of bending strength, flexural rigidity, sheath presence, and genotype (Model 7)*

| Model | Table 5: $\sqrt{Strength}$ | Df | Sum Sq. | Mean Sq. | F-value | P-value |
| --- | --- | --- | --- | --- | --- | --- |
| (6) | $\sqrt{Flexural Rigidity}$ | 1 | 10308.4 | 10308.4 | 990.6 | <2.2e-16 |
|  | Sheath Presence | 1 | 1117.9 | 1117.9 | 107.4 | <2.2e-16 |
|  | Wheat Type | 1 | 303.6 | 303.6 | 29.2 | 1.0e-07 |
|  | Residual | 492 | 5119.8 | 10.4 |  |  |
| (7) | $\sqrt{Flexural Rigidity}$ | 1 | 10308.4 | 10308.4 | 2179.3 | < 2.2e-16 |
|  | Sheath Presence | 1 | 1117.9 | 1117.9 | 236.3 | < 2.2e-16 |
|  | Genotype | 30 | 3233.4 | 107.8 | 22.8 | < 2.2e-16 |
|  | Residual | 463 | 2190.0 | 4.7 |  |  |

*Table 4: Multinomial logit regression of failure type, sheath presence, and wheat type*

| $Failure mode$ | Predictor | Estimate | Std. Error | P-value |
| --- | --- | --- | --- | --- |
| Crushed | Intercept | -0.57 | 0.19 | 3.15e-03 |
|  | Sheath Presence | 1.24 | 0.19 | 8.21e-11 |
|  | Wheat Type | -0.03 | 0.20 | 0.87 |
|  |  |  |  |  |
| Splintered | Intercept | -3.68 | 0.68 | 6.69e-08 |
|  | Sheath Presence | 1.75 | 0.68 | 0.01 |
|  | Wheat Type | -0.44 | 0.61 | 0.47 |
